# Special Analytical and Health Commission on Tinned and Preserved Food

**Published:** 1906-08-04

**Authors:** 


### SPECIAL ANALYTICAL AND HEALTH COMMISSION ON TINNED AND PRESERVED FOOD.

### VI. "SPECIAL" CANNED MEAT FOODS.

In our last article we completed the consideration of canned and preserved beef and mutton. We now propose to consider what may be termed "special" canned and preserved meat foods. The first article coming under this head is tongue. Tongue may be regarded as one of the most favoured special parts of an animal from the gastronomic standpoint, and certainly one which lends itself most readily to the preserving art. Tongue has from classical times been regarded as a delicacy, and it will doubtless be remembered by those who are acquainted with the menus of ancient Roman feasts that larks' tongues were one of the *bon bouches* with which the lengthy monotony of Roman banquets were relieved. The the tongue of the lark was supposed, according to ancient lore, to give especially sweet qualities to the voice of the consumer. Modern dietetics, more practical, if less refined, has hit upon the tongue as a viand to be encouraged for less idealistic reasons. The tongue is essentially muscle-flesh, although with it is generally incorporated the salivary glands, and, speaking generally, it partakes of the special qualities of the flesh of the animal to which it belongs. For instance, calves' tongue partakes of the peculiar properties of veal—namely, it is of pale colour and less consistent than beef, and, while containing less iron and alkali salts than the former, is richer in connective tissue. The tongue of the sheep is less firm than that of the ox; its fat is whiter, has a different melting-point, and both fat and lean have a more distinctive odour. The characteristic, however, that distinguishes the muscular tissue forming the tongue of any animal from that of the other muscles of the same animal is very important from the gastronomic standpoint, and is the fact that tongue-flesh is less tough than any other. The relative toughness of meat depends upon the proportion of white (collagenous) connective tissue fibres it contains as compared with elastic tissue fibres. A most ingenious apparatus has been invented by Lehmann for estimating the relative toughness of the different edible parts of animals, and he has found that whereas collagenous fibres require 1,040 grammes to rupture them, elastic tissue requires only 500. Measuring toughness on this scale a boiled fillet of beef may be regarded as having a toughness of 84, whereas a boiled tongue has a toughness of 64. The tenderest parts of an animal, speaking generally, are the glandular structures, the brain, for instance, having a toughness of 2.4. It is interesting in this connection to note that game on hanging for three days loses 25 per cent. of its toughness. We have not thought it necessary to draw attention to the fact that toughness is *cæteris paribus* a measure of digestibility. The individual fibres of any meat food have eventually to be separated, ruptured, and disintegrated by the digestive juices, and the resistance which they offer to mechanical strain is a measure of that which they will offer to digestibility. In the table before us we cannot find that the toughness of sweetbread has ever been measured, but it probably lies between that of liver (which is 8) and brains (which, as we have seen, is 2.4). If for a moment we consider these figures, they will give us useful indications for the order in which we should put an invalid with a weak digestion upon animal foods; and, further, they will explain what its chemical composition does not—namely, the reputation that tongue has as a light breakfast dish.

Canned or preserved tongue is, as a rule, both cooked and pickled. The details of these processes it is beside our function to discuss. Suffice it to say that saltpetre is a constant ingredient, and that the red appetising colour of good tongue should be entirely due to the chemical reaction of this substance upon the hæmoglobin of the muscle. The average composition of canned tongue is as follows: water, from 35 per cent. to 65 per cent.; nitrogenous matter, 15 per cent. to 25 per cent.; fat, 15 per cent. to 30 per cent.; ash, 3 per cent. to 8 per cent. The variation in these figures is due essentially to the degree of drying or smoking, which varies very much in different processes, but an average water content of fresh sheep's tongue is 67.44 per cent., and that of fresh ox tongue is 63.8 per cent. Another characteristic of tongue is that its fat content is relatively high, and that the fat exists in a state of fine mechanical division which renders it digestible. Tongue is subject, however, to many diseases and pathological states. We may mention *inter alia* that it is not uncommonly attacked by the so-called ray-fungus, which produces the disease known as actinomicosis. Tongue flesh seems, too, to be particularly liable to decomposition, and in this connection it is interesting to note that bad tinned pig's tongue was the cause of a serious outbreak of poisoning at Oldham. Canned tongue should possess a good and characteristic flavour; the fat should be of good colour, and not rancid in taste; the meat should be firm, of a good red colour, and should carve well. No gas should escape upon opening the tin.

### Samples Received.

We have received from the Liebig Extract of Meat Company a specimen of their canned prime Fray Bentos ox tongue. This Company, as they do not use the tongue of the animal for making their extract of meat, naturally have a tremendous supply of ox tongues. Every animal passes expert veterinary examination before use. We have examined this article, and find it to be in every way recommendable. Messrs. Aplin Barrett, Limited, of Yeovil, have sent us a tin of their St. Ivel's calves' tongues. The contents was of good texture and light calf tongue colour, and possessed a good flavour. We have received from Watling and Sons a specimen of their English rolled ox tongues preserved in glass. These tongues are exceedingly well cured, and possess a most admirable taste and flavour. The curing is perhaps a trifle stronger than the average tongue we have examined, but many consumers will prefer this. We put this tongue to a severe test with regard to its keeping qualities after being opened, and it remained good and moist for a length of time ample to fulfil ordinary domestic use. Messrs. Travers and Company sent us a tin of selected lunch tongue (Ship brand). This article is packed under Government supervision, and is certified by inspectors under the Live Stock and Meat Export Act of 1895. We regard it as an exceedingly good preparation. Messrs. Palethorpe have also submitted to us a tin of their lunch tongues. This article is well up to the usual standard of this firm, and the public can have every confidence that it is of the best quality in every way. Messrs. P. Winter and Company have forwarded a tin of their preserved sheeps' tongues. These tongues are imported from France, and are of exceptionally good quality. They possess a piquant and delicate flavour, and appear to us to be especially suitable for invalid use. We are indebted to Messrs. Cosenza and Company, of Wigmore Street, the well-known Italian warehousemen, for a specimen of their sheeps' tongues à la sauce Ravigotte. This preparation is intended to be eaten hot, and the directions are that the tin should be boiled in water for fifteen minutes. To those who like the flavour of shallots this makes a most savoury dish, and one most easily prepared, as boiling water is all that is needed. Our experience of the tongues contained in this preparation of Messrs. Cosenza is that they are tender, of good colour, texture, and taste. We may conclude our remarks upon canned and preserved tongue by saying that although we have examined a large number of samples of this commodity, we have not found one anything but thoroughly good and wholesome, and we consider the favour placed by the public in this article of diet to be thoroughly justified and to be encouraged.

